# Validity of self-reported sleep duration in the Cancer Prevention Study– 3

**DOI:** 10.1371/journal.pone.0307409

**Published:** 2024-08-16

**Authors:** Sidney M. Donzella, Matthew Masters, Amanda I. Phipps, Alpa V. Patel, Charlie Zhong

**Affiliations:** 1 Department of Epidemiology, University of Washington, Seattle, WA, United States of America; 2 Department of Population Science, American Cancer Society, Atlanta, GA, United States of America; 3 Public Health Sciences Division, Fred Hutchinson Cancer Center, Seattle, WA, United States of America; Mexican Social Security Institute: Instituto Mexicano del Seguro Social, MEXICO

## Abstract

**Purpose:**

We examined the one-year test re-test reliability and validity criterion of survey-assessed sleep duration collected from two separate questions.

**Methods:**

The Activity Validation Sub Study included 751 participants of the Cancer Prevention Study-3 study to further investigate rest/activity cycles. Sleep duration was collected using three methods: survey, Daysimeter device, and sleep diary. Survey-assessed sleep duration was collected using 2 different questions, each with different response options (categorical and continuous). Selected participants (n = 170) were asked to wear a Daysimeter device for seven consecutive days for two non-consecutive quarters. Participants were excluded from the current study due to incomplete/implausible survey or device data or reported working night shift. We calculated reliability of pre- and post-survey sleep duration for both survey question using Spearman correlation. We used the method of triads to estimate the validity coefficient (VC) between the three sleep duration measurements in the present study and the “true” latent sleep duration measure, and bootstrapping methods to calculate the 95% confidence intervals (95%CI).

**Results:**

Of 119 participants included in the study (52.10% male), test-retest correlation showed strong and moderate correlations for sleep duration collected continuously and categorically, respectively. The VC for survey-assessed continuous sleep duration was 0.82 (95%CI 0.71, 0.90) for weekday and 0.68 (95%CI 0.46, 0.83) for weekend. Performance of the VC was slightly weaker for survey-assessed categorical sleep duration (weekday VC = 0.57 95%CI 0.42, 0.71; weekend VC = 0.47 95%CI 0.29, 0.62).

**Conclusion:**

The two survey-assessed sleep duration questions used in the AVSS and CPS-3 cohorts are valid approximations of sleep duration.

## Introduction

The field of sleep epidemiology has grown in the past several decades. Many epidemiologic studies have suggested a relationship between sleep duration and various health outcomes, including mortality [1]. Such epidemiologic studies often rely on self-reported sleep measures, which carry significantly lower research costs and minimize participant burden relative to more objective measures (e.g., actigraphy). Although self-reported sleep measures are widely used in epidemiology research, these measures may be influenced by participant perception of sleep [2], how the question is worded or formatted [3], and/or various preexisting health conditions [4], all of which may potentially lead to systematic or random error in the measurement. Reliable and valid assessment of sleep duration is necessary to accurately capture sleep patterns and evaluate the impact of sleep on health.

Often, the validity of subjective sleep measures is assessed in a two-way comparison using accelerometry [5] or polysomnography [6] (PSG) as the comparator. Accelerometers are a non-invasive technique widely used to objectively measure sleep duration through movement [7]. Although accelerometry provides objective sleep measures, the collection and processing of these data is imperfect [7, 8] and may under- or over-estimate sleep duration compared to polysomnography (PSG) [7]. PSG is the gold standard of objective sleep measurement; however, its use in large-scale epidemiologic studies is generally infeasible given that it is resource, time, and cost intensive. Therefore, the use of a triangular comparison (method of triads) between questionnaire, reference, and objective measures may be more appropriate to quantitatively describe the validity of sleep collected via questionnaire in large prospective cohort studies.

The objective of this study was to 1) assess the one-year test-retest reliability of sleep duration collected from two different questionnaire items and 2) examine the criterion validity of self-reported sleep duration collected from two different questionnaire items using the method of triads, in a validation sample of participants enrolled in a large, nationwide, prospective cohort study.

## Methods

### Study population and design

The American Cancer Society Cancer Prevention Study-3 (CPS-3) is a prospective cohort study of over 300,000 US adults with the primary goal of investigating cancer incidence and mortality [9]. Participants aged 35 to 65 years with no previous cancer history (except for basal and squamous cell skin cancer) were recruited and enrolled from 2006 to 2013. Of the 303,682 participants enrolled, approximately 254,000 completed the baseline questionnaire and were sent subsequent follow-up questionnaires. The CPS-3 study recruitment and design has been previously described in detail [9].

The CPS-3 Activity Validation Sub Study (AVSS) was conducted in 2015 to further investigate rest/activity cycles among a subset of CPS-3 study participants [10]. A total of 10,000 CPS-3 participants were invited to participate in the AVSS, with sampling stratified by sex, race, and ethnicity. A total of 1,801 participants accepted the invitation and 751 participants who completed the 2015 CPS-3 follow-up survey were enrolled in the AVSS. A subsample of AVSS participants (N = 190) were invited to participate in the collection of light exposure and objective sleep measures. The CPS-3 study and the AVSS were approved by the Emory University Institutional Review Board. All CPS-3 participants provided written consent when they completed the on-site survey at enrollment with trained volunteers serving as the witness. AVSS participants provided consent electronically with no witness present. No minors were enrolled in the CPS-3 or AVSS studies.

The AVSS prospectively collected data from July 27, 2015 to October 26, 2016. At baseline, participants were asked to complete a “pre-survey” questionnaire that collected information on sleep patterns as well as other demographic and lifestyle factors. Participants completed various objective and subjective sleep measurements throughout the duration of the study. At the end of the AVSS study, participants completed the same questionnaire which we refer to as the “post-survey” questionnaire.

### Sleep measures

#### Survey

Sleep duration was collected using 2 different survey questions. The first question asked participants “During the past year, estimate the hours per day you spent sleeping on typical weekdays and weekends” with possible hours/day response categories being “0, <1, 1–2, 3–4, 5–6, 7–8, 9–10, 11+”. Weekdays were defined as Sunday-Thursday night and weekends included Friday and Saturday nights. The midpoint for each response category was used as the absolute sleep duration, and 12 was used as the midpoint for the 11+ category. This measure will be referred to as survey-assessed categorical sleep duration.

The second sleep duration question was as follows: “During the past year, what time do you typically try to go to sleep and wake up?”. Participants were asked to fill in time they fell asleep and time they woke up for workday and non-workday, separately, in the format of hh:mm, and to mark AM or PM. All responses were converted to 24:00 time and the following assumptions were made: 1) if hour asleep was between 1:00–5:59 and AM/PM was not selected, then time asleep was assumed to be 01:00–05:59; 2) if hour asleep was between 6:00–11:59 and AM/PM was not selected, then time asleep was assumed to be 18:00–23:59; 3) if hour asleep was 12, then time asleep was assumed to be midnight regardless of AM/PM selection. Due to limited data on occupation schedule, workday and non-workdays were assumed to correlate with weekday and weekends, respectively. This measure will be referred to as survey-derived continuous sleep duration.

#### Accelerometer

Selected participants (n = 190) were asked to wear a Daysimeter device for seven consecutive days for two non-consecutive quarters (Q1/Q3 or Q2/Q4) [11]. Data collection throughout the course of seven consecutive days provides the opportunity to observe potential differences in weekday and weekend sleep patterns. Similarly, we asked participants to wear the Daysimeter during two non-consecutive seasons to account for potential seasonal differences in sleep that may occur [12, 13]. The Daysimeter has an accelerometer embedded within the device [14] and was worn on the non-dominant wrist during sleeping hours. Raw Daysimeter accelerometry data were processed and assigned an activity index. The sleep algorithm was developed based on the Actiware-Sleep Version 3.4 algorithm [15]. Date of wear was extracted from the raw Daysimeter data and used to determine type of day (weekday or weekend). Valid device data was determined to be at least one quarter of 3 weekdays + 1 weekend of wear. Average sleep duration estimates for valid weekdays and weekends were calculated separately.

#### Sleep diary

Participants were asked to complete a sleep diary for every day of Daysimeter wear. In the sleep diary, participants were asked “What is the best estimate of how much actual sleep you got last night?” Participants estimated the number of hours and minutes of their actual sleep duration for each night. Corresponding diary data for each day of Daysimeter data was required for inclusion in final study population. Diary data were averaged for weekdays and weekend separately.

### Exclusion criteria

Of the 190 AVSS study participants assigned a Daysimeter, participants were excluded from the current analysis for the following reasons: incomplete AVSS pre- or post-survey data (n = 15), implausible device data (n = 1), reported working night shift (n = 7), insufficient days of device wear or diary entries (n = 40), or reported less than 3 hours or greater than 14 hours of sleep duration for any of the sleep measures (n = 8). Thus, final analyses were conducted on a sample of n = 119 participants.

## Statistical analysis

Mean sleep duration was calculated separately based on the two survey questions, the Daysimeter, and the diary. Reliability of survey-reported sleep duration was calculated using Spearman correlation of the pre- and post-survey for each survey question (categorical and continuous) overall.

**Fig 1:**
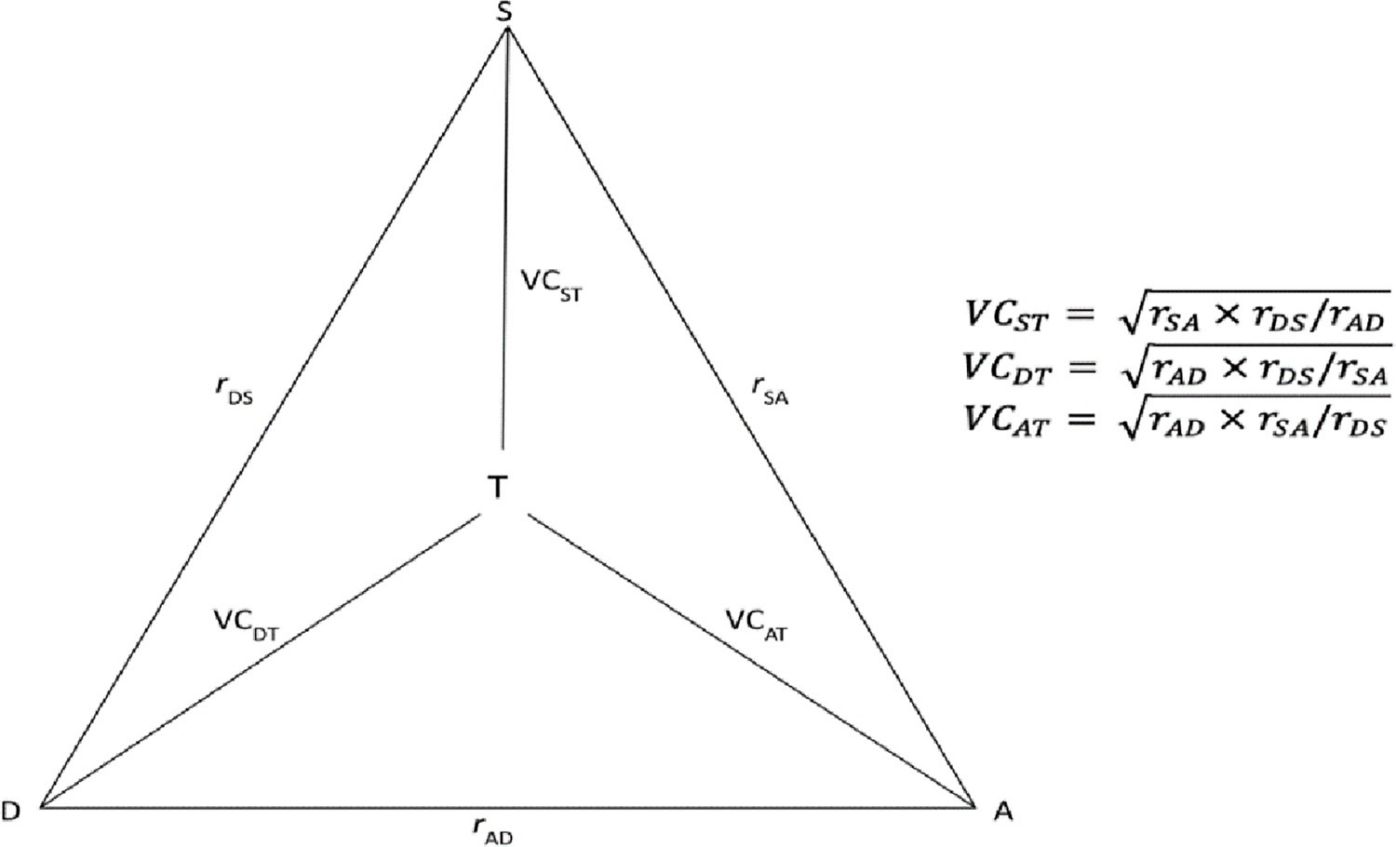
Illustration of the method of triads used to estimate the validity coefficients (VC_ST_, VC_DT_, VC_AT_) between true sleep duration (T) and the post-survey (S), diary (D), and accelerometer (A). Pairwise correlation coefficients (*r*_SA_, *r*_DS_, *r*_AD_) between the different methods (S, D, A) are used to calculate the VCs.

We used the method of triads [16] to estimate the validity coefficient (VC) between the three sleep duration measurements in the present study and the “true” latent sleep duration measure. The method of triads is a technique in factor and path analysis that uses an observed correlation matrix to fit a theoretical correlation matrix [16]. This approach assumes a positive linear relationship between the observed and true measures and that the random errors in each observed estimate are independent [16]. In practice, the “true” sleep duration measurement is not available. The method of triads concludes that the any observed associations between the three measures being compared is due to their relationship with the “true” latent sleep duration [16]. A VC is calculated for the observed measures with the “true” sleep duration using a set of Pearson pairwise correlation coefficients (*r*) (Fig 1). Correlations were interpreted following standard convention (<0.40: weak, 0.40–0.69: moderate, 0.70–0.89: strong, and ≥0.90: very strong) [17]. We used bootstrapping methods with a sample size of n = 1,000 to calculate the 95% confidence intervals (CI). All analyses were done in R version 4.2.3.

## Results

A total of 119 participants were included in the study, among whom 52.10% were male (n = 62) and the mean age was 51.74 years (SD = 9.71, range 32–73 years) (Table 1). Over half of the participants self-reported race as White (61.34%). The majority of participants reported average sleep quality to be “fairly well” or “very well” (88.98%) and reported having not taking sleep medicine in the past month (86.21%) (Table 2). Average weekly sleep duration was shortest when measured by the diary (412.09 min, SD = 54.67 min) and the difference between average weekday and weekend sleep duration ranged from approximately 32–58 minutes across the various sleep duration measures.

**Table 1:** Baseline characteristics of included AVSS participants, n = 119.

	N (%) or Mean (SD)
Age	51.74 (9.71)
Sex	
Male	62 (52.10%)
Female	57 (47.90%)
Race	
White	73 (61.34%)
Black	22 (18.49%)
Hispanic	24 (20.17%)
Education	
< = HS	3 (2.52%)
Some college or 2yr	24 (20.17%)
4-yr college degree	41 (34.45%)
Graduate degree	34 (28.57%)
Missing	17 (14.29%)
Marital Status	
Single	12 (10.08%)
Married/Partnered	90 (75.63%)
Divorced/Sep./Widowed	16 (13.45%)
Unknown	1 (0.84%)
BMI at Follow-up	27.28 (5.73)
Employment status at FU	
Not Employed	18 (15.38%)
Employed	99 (84.62%)
Unknown	2 (1.68%)
Physical activity guidelines^a^	
Meets	98 (82.35%)
Does not meet	21 (17.65%)

Abbreviations: SD, standard deviation; BMI, Body mass index

^a^ Physical Activity Guidelines of ≥7.5 MET/hrs of moderate to vigorous activity per week

**Table 2:** Sleep characteristics of AVSS participants, n = 119.

	All	Survey-derived^a^ continuous	Survey-assessed^a^ categorical	Accelerometer	Diary
Mean overall sleep duration, minutes	-	456.55 (53.43)	448.74 (61.92)	425.45 (53.54)	412.09 (54.67)
Mean weekday sleep duration, minutes	-	440.10 (56.20)	434.62 (68.08)	415.61 (55.32)	402.86 (57.03)
Mean weekend sleep duration, minutes	-	497.68 (74.19)	484.03 (69.75)	450.05 (70.82)	435.16 (68.13)
Sleep duration guidelines^b^	-		-		
Does not meet	-	23 (19.33%)	48 (40.34%)	48 (40.34%)	61 (51.26%)
Meets	-	96 (80.67%)	71 (59.66%)	71 (59.66%)	58 (48.74%)
Sleep quality		-	-	-	-
Very well	47 (39.83%)	-	-	-	-
Fairly well	58 (49.15%)	-	-	-	-
Fairly poor	13 (11.02%)	-	-	-	-
Very poor	0 (0.00%)	-	-	-	-
Unknown	1 (0.84%)	-	-	-	-
Use of sleep medicine in past month		-	-	-	-
No	100 (86.21%)	-	-	-	-
Yes	16 (13.79%)	-	-	-	-
Missing	3 (2.52%)	-	-	-	-
Chronotype		-	-	-	-
Def. Morning	39 (33.05%)	-	-	-	-
More Morning	31 (26.27%)	-	-	-	-
More Evening	22 (18.64%)	-	-	-	-
Def. Evening	17 (14.41%)	-	-	-	-
None	9 (7.63%)	-	-	-	-
Unknown	1 (0.84%)	-	-	-	-

Abbreviations: SD, standard deviation; BMI, Body mass index

^a^ Post-survey measures

^b^ National Sleep Foundation sleep duration recommendations of 7–9 hours per night

Test-retest correlation for the pre- and post-survey showed moderate and strong correlations for sleep duration collected categorically and continuously, respectively (Table 3). Bivariate Pearson correlations between the survey, Daysimeter, and diary are shown in Table 4. Correlations of sleep duration showed moderate to strong agreement between measures with the exception of categorical weekend sleep duration collected from the survey and the Daysimeter (*r* = 0.36, 95% CI 0.20–0.51). Generally, survey-derived continuous sleep duration had better correlations with the Daysimeter and diary compared to the survey-assessed categorical sleep duration.

**Table 3:** Spearman correlation for pre- and post-survey sleep duration, n = 119.

	Survey-derived continuous sleep duration ρ (95% CI)		Survey-assessed categorical sleep duration ρ (95% CI)	
	Weekday	Weekend	Weekday	Weekend
All	0.81 (0.71–0.87)	0.77 (0.67–0.85)	0.60 (0.43–0.74)	0.69 (0.51–0.80)

Abbreviations: CI, confidence interval

**Table 4:** Pearson correlation coefficient for post-survey sleep duration, Daysimeter, and diary, n = 119.

	Survey vs Accelerometer *r*_*SA*_ (95% CI)		Survey vs Diary *r*_*SD*_ (95% CI)		Accelerometer vs Diary *r*_*AD*_ (95% CI)	
	Weekday	Weekend	Weekday	Weekend	Weekday	Weekend
**Survey-derived continuous sleep duration**						
All	0.79 (0.71–0.85)	0.59 (0.46–0.69)	0.67 (0.55–0.76)	0.59 (0.46–0.70)	0.79 (0.71–0.85)	0.77 (0.68–0.83)
**Survey-assessed categorical sleep duration**						
All	0.51 (0.36–0.63)	0.36 (0.20–0.51)	0.51 (0.36–0.63)	0.46 (0.30–0.59)	0.79 (0.71–0.85)	0.77 (0.68–0.83)

Abbreviations: CI, confidence interval

The VC for survey-derived continuous sleep duration and the latent sleep duration was 0.82 (95% CI 0.71, 0.90) for weekday and 0.68 (95% CI 0.46, 0.83) for weekend (Table 5). Performance of VC was slightly weaker for survey-assessed categorical sleep duration (weekday VC = 0.57 95% CI 0.42, 0.71; weekend VC = 0.47 95% CI 0.29,0.62).

**Table 5:** Validity coefficient for sleep duration using method of triads, n = 119.

	Survey-derived continuous sleep duration VC_SD_^a^ (95% CI)		Survey-assessed categorical sleep duration VC_SD_^a^ (95% CI)	
	Weekday	Weekend	Weekday	Weekend
All	0.82 (0.71–0.90)	0.68 (0.46–0.83)	0.57 (0.42–0.71)	0.47 (0.29–0.62)

Abbreviations: CI, confidence interval; VC, validity coefficient; SD, sleep duration

^a^ validity coefficient of post-survey sleep duration and true latent sleep duration

## Discussion

This study used data collected from a year-long activity validation study to assess how well two different survey questions measured average sleep duration. Survey-derived continuous sleep duration performed better than survey-assessed categorical sleep duration in the test-re-test Spearman correlation and the VC. Overall, survey-based sleep duration showed moderate to strong reliability with latent sleep duration, suggesting that self-reported sleep duration is a reliable measure of actual sleep duration.

Performance of survey-based sleep duration varied by question design. There was stronger agreement between continuously derived sleep duration and latent sleep duration compared to categorical sleep duration. This may be due to the categorical response options capturing an approximated rather than a more precise sleep duration. Categorical response options included in the final analyses captured a two-hour range in sleep duration, with the exception of the last category, leading to the midpoint of each category to vary by two hours. For example, if a participant reported 6.5 hours on the pre-survey (categorical option: 5–6 hours) and 7 hours on the post-survey (categorical option: 7–8 hours), the Spearman correlation for the categorical response would compare 6 hours versus 8 hours and 6.5 hours versus 7 hours for the continuous response. This difference in precision is displayed in the Spearman correlations for pre- and post-survey sleep duration where categorical sleep duration had lower correlations than continuous sleep duration. Twenty-eight and 23 participants selected a different categorical option from the pre-survey to post-survey on weekdays and weekends respectively, which would have contributed to the reduced agreement in the correlation. Although categorical sleep duration still showed moderate agreement with latent sleep duration, the varied performance between categorically- and continuously-derived sleep duration supports the notion that the structure of the question and response impacts the reliability of the measure.

We examined sleep duration on the weekday and weekends separately to investigate if the self-reporting of sleep duration changes based on type of day. The measurement of survey-assessed weekday sleep duration generally appeared to be more consistent and in better agreement with latent sleep duration compared to survey-assessed weekend sleep duration, consistent with previous studies [18, 19]. Sleep patterns often vary throughout the week, commonly due to work and social commitments [20]. The accumulation of sleep debt from insufficient sleep on weekdays can lead to longer sleep bouts on the weekends to “catch-up” on sleep [20]. This phenomenon was seen in our study as mean weekend sleep duration was greater than mean weekday sleep duration across all measurement tools. It is possible that the self-reporting of average weekend sleep duration presents more challenges compared to average weekday sleep duration due to less structured and/or inconsistent weekend schedules. In addition, we were limited to just two weekends of observations, which may produce additional variability. Results highlight the importance of measuring sleep duration separately on weekdays and weekends as mean values and reliability of measurements may vary.

Prior studies have used two-way comparisons to assess the reliability of sleep duration measures [5, 6, 21, 22]. Some have compared survey-assessed sleep duration to objective measures such as actigraphy or PSG and reported poor correlations [6, 19], while others have compared survey-assessed sleep duration to sleep diaries and have reported correlations ranging from 0.39 to 0.48 [18]. Although the use of two-way comparisons have been helpful to understand how survey-assessed sleep performs relative to other subjective or objective tools, no single sleep measurement tool is without error making it difficult to select a reference tool. It is possible that the random errors of the measurements used in a two-way comparison are correlated which violates the assumptions needed for a traditional validation approach [16]. The method of triads can be helpful when there is no perfect measure to serve as a reference, provided the two model assumptions are met; that there is a linear relationship between the measures and independence of errors [16]. With the addition of the third measurement in the method of triads, and the assumption of a positive linear relationship between the observed and true measures, we were able to overcome this limitation. In our study, we could not rule out the possibility of a correlation between the random errors of the survey and diary, but we believe the linear assumption will hold. As a result, the estimated VCs of the two survey-assessed sleep duration measures with latent sleep duration should be viewed as the upper limit of criterion validity [16]. To our knowledge, this is the first study to assess criterion validity of survey-assessed sleep duration using the method of triads.

Differences in structure of survey questions and sleep diaries may impact participant responses, reproducibility, and validity. Sleep diaries require participants to recall sleep duration from only the previous night while survey questions may require recall of average sleep duration over the prior year. Cognitive burden of the sleep diary is lower than that of the survey. Additionally, sleep is dynamic and varies throughout the duration of a year. Asking participants to recall the average sleep duration during the prior year may introduce non-differential measurement error. As previously mentioned, categorical versus continuous response options may lead to varying precision of measurement introducing measurement error. The sleep diary used in this study asked participants to report an estimate of “actual sleep” and the average duration recorded from the sleep diary was lower than that recorded from both survey questions. It is possible that when answering the diary question participants removed time from nocturnal awakening. This would be particularly important to consider when working with populations at higher risk of experiencing insomnia symptoms (e.g., cancer survivors [23]). Careful questionnaire and diary design may reduce random or systematic error in the measurements.

Strengths of this study include the diverse study population, as 18% and 20% of study participants self-reported their race as Black and Hispanic, respectively, and the ability to leverage multiple measures of sleep duration. We were also able to capture seasonality due to the collection of Daysimeter sleep measures in two non-consecutive quarters. Our study is not without limitations. Daysimeter devices were sent to only a small proportion of AVSS participants leading to a relatively small sample size after applying the exclusion criteria. Measures of accelerometer-measured sleep may change due to the device and/or algorithm used [24, 25]. The use of the Daysimeter device as the accelerometer limits the generalizability to other studies as Actigraph devices are the most commonly used research-grade device and utilize different data-processing algorithms. However, the Daysimeter has been validated for research [14], and the method of triads seeks to address such limitations [16]. Another limitation of this study is the relatively long test-re-test period. Sleep is dynamic and it is possible that sleep patterns can naturally change over the course of a year. Despite the long test-re-test period, correlations for pre- and post-survey measures remained moderate to strong. Due to the relatively small sample size, we were unable to stratify by various factors that may impact sleep patterns such as age, sex, chronotype, and use of sleep medicine. Future research should consider the impact of various demographic, behavioral, and health factors on the validation of sleep measures. Lastly, the AVSS study population is representative of the ACS CPS-3 cohort, but not of the more general US population.

## Conclusion

Criterion validity was stronger for weekday sleep duration measures compared to weekend sleep duration. The two survey-assessed sleep duration questions used in the AVSS and CPS-3 cohorts displayed acceptable reliability and validity when using the method of triads to assess sleep duration.
